# Plasma-Etched Black GaAs Nanoarrays with Gradient Refractive Index Profile for Broadband, Omnidirectional, and Polarization-Independent Antireflection

**DOI:** 10.3390/nano14131154

**Published:** 2024-07-06

**Authors:** Yi-Fan Huang, Yi-Jun Jen, Varad A. Modak, Li-Chyong Chen, Kuei-Hsien Chen

**Affiliations:** 1 Department of Mechanical Engineering, National Chin-Yi University of Technology, Taichung 411030, Taiwan; 2 Department of Electro-Optical Engineering, National Taipei University of Technology, Taipei 106, Taiwan; 3 Institute of Atomic and Molecular Sciences, Academia Sinica, Taipei 10617, Taiwan; 4 International Graduate Program of Molecular Science and Technology, National Taiwan University (NTU-MST), Taipei 10617, Taiwan; 5 Molecular Science and Technology Program, Taiwan International Graduate Program (TIGP), Academia Sinica, Taipei 11529, Taiwan; 6 Department of Physics, National Taiwan University, Taipei 10617, Taiwan; 7 Center of Atomic Initiative for New Materials, National Taiwan University, Taipei 10617, Taiwan; 8 Center for Condensed Matter Sciences, National Taiwan University, Taipei 10617, Taiwan

**Keywords:** black GaAs NTs, gradient refractive index, polarization-independent antireflection

## Abstract

Black GaAs nanotip arrays (NTs) with 3300 nm lengths were fabricated via self-masked plasma etching. We show, both experimentally and numerically, that these NTs, with three gradient refractive index layers, effectively suppress Fresnel reflections at the air–GaAs interface over a broad range of wavelengths. These NTs exhibit exceptional UV-Vis light absorption (up to 99%) and maintain high NIR absorption (33–60%) compared to bare GaAs. Moreover, possessing a graded layer with a low refractive index (n = 1.01 to 1.12), they achieve angular and polarization-independent antireflection properties exceeding 80° at 632.8 nm, aligning with perfect antireflective coating theory predictions. This approach is anticipated to enhance the performance of optoelectronic devices across a wide range of applications.

## 1. Introduction

Gallium arsenide (GaAs), a direct bandgap semiconductor material with high absorption coefficients, has been extensively utilized in semiconductor optoelectronics for applications such as solar cells [1,2], lasers [3], and light-emitting diodes [4,5]. The primary issue for this type of application is the high refractive index of GaAs (n = 3.8), leading to surface reflectance exceeding 35% across the visible spectrum. The application of antireflective coating layers on these optoelectronic devices offers an efficient solution to enhance light absorption efficiency. Single-layer or multi-layer antireflection coatings can typically reduce surface reflectance to nearly zero for light of a certain wavelength range and angle of incidence (AOI). Typical designs of traditional antireflection coatings (the optical thickness of each layer is at least one-quarter of the wavelength) are often ineffective at suppressing reflectance over a broad spectral band and a wide range of AOIs.

GaAs materials featuring subwavelength [6,7,8,9] or antireflective structures [10,11,12,13,14,15,16] that exhibit nearly zero reflectance are referred to as “black GaAs”. Black GaAs is preferred for antireflective applications due to its black appearance and exceptional ability to trap light effectively across a wide spectral and AOI range [6,7,8,9,10,11,12,13,14,15,16]. For instance, GaAs subwavelength structures exhibit an average reflectance below 5% across wavelengths ranging from 350 to 900 nm [6]. Additionally, these structures have demonstrated the ability to maintain low reflectance (~5%) for AOIs up to 60° for both p- and s-polarizations at a wavelength of 632.8 nm [6]. Various mask- or self-mask-based etching techniques, such as inductively coupled plasma etching [6,7,8,9,10,15,16] and metal-assisted chemical etching [11,12,13,14], have been employed to fabricate black GaAs structures. Different types of black GaAs surface morphologies, including nanocones [6,7,8,9], nanograsses [10], nanopores [11], nanowires [13,16], and micrometer-scale structures [12,14], have been demonstrated to diminish Fresnel reflection across a wide spectrum of wavelengths and AOIs. Furthermore, black GaAs has been successfully utilized in III-V solar cells [17,18], photodetectors [19], and photo-electrochemical hydrogen generation [20] in recent years.

The reduced reflection from a black GaAs surface occurs because light no longer encounters a distinct boundary between the air and the substrate material. Typically, reflections primarily occur at changes in the refractive index, and the elimination of the sharp boundary between the air and material interfaces significantly diminishes reflections. Therefore, the broadband antireflective behavior of black GaAs surfaces is thought to be analogous to the moth-eye effect [21,22,23]. Moth eyes possess a natural nanostructure on their surface that creates a gradient in the refractive index (GRI) from the air to the material of the eye [21,22,23]. These nanostructures can significantly reduce surface reflections over a wide spectral and AOI range [21,22,23]. Thus, to validate the broadband, wide-angle, and polarization-independent antireflection properties of black GaAs, it is imperative to confirm the presence of a gradient change in the refractive index profile from air to the GaAs substrate. However, the numerous instances of black GaAs reported in the literature over the past decade have primarily focused on experimental results related to antireflection performance [6,7,8,9,10,11,12,13,14,15,16]. To the best of our knowledge, no reports have documented experimental evidence of actual GRI changes in black GaAs.

In this study, we utilize the self-masked dry etching (SMDE) technique in an electron cyclotron resonance (ECR) plasma system to produce black GaAs with nanotip (NT) arrays on a bare GaAs substrate. Our primary focus is on measuring reflectance spectra and simulating the GRI profile of black GaAs NTs to confirm that they achieve excellent broadband, omnidirectional, and polarization-independent antireflective properties. These measurements encompass hemispherical reflectance and angular dependence of specular reflectance across the UV-Vis-NIR wavelength range, as well as s- and p-polarized reflectance at 632.8 nm. Furthermore, our optical modeling of the GRI profile for black GaAs NTs, involving three graded layers, concurs with the measured angular dependence of polarization reflectance data at 632.8 nm. Black GaAs NTs, characterized by exceptional optical properties, promise significant enhancements in various optoelectronic and photovoltaic devices, including photodetectors, solar cells, and photocathodes, especially for solar hydrogen production.

## 2. Materials and Methods

### 2.1. Sample Preparation

The black GaAs NT samples were fabricated using the SMDE technique in a high-density ECR plasma system. A detailed description of the formation of aperiodic nanostructured arrays via the SMDE technique can be found in our previously published papers [24,25]. A brief description of the process is provided below. Before the actual NT fabrication process, single-crystal (100), undoped, and one-sided polished GaAs wafers were cleaned with acetone, methanol, and deionized water sequentially. The ECR plasma etching process for the SMDE technique was conducted under a microwave power of 1200 W, a pressure of 3.2 mTorr, and a gas flow ratio of methane (CH_4_)/silane (SiH_4_)/argon (Ar)/hydrogen (H_2_) of 3:0.2:5:8 sccm. The total ECR plasma etching process lasted for 16 h. In the etching process of SMDE technology, the length of nanotips can be controlled by adjusting the plasma etching duration. For instance, GaAs nanotips with a length of approximately 380 nm can be produced with an etching time of three hours, while those with a length of about 830 nm require six hours of etching, as shown in Supporting Figure S1. Additionally, as reported in our previous study [25], the process temperature in SMDE technology also influences the nanotip size; specifically, the length of the nanotip decreases as the process temperature increases.

### 2.2. Material Characterization and Optical Measurements

The morphology and dimensions of the black GaAs nanostructures were analyzed using a JEOL 6700 (Tokyo, Japan) field emission scanning electron microscope (SEM). The antireflection properties of untreated GaAs and black GaAs samples were assessed using three different optical measurement systems. The hemispherical reflectance (R%), hemispherical transmittance (T%), and hemispherical absorbance (A% = 100% − R% − T%) of all GaAs samples were measured in the wavelength range from 300 nm to 2000 nm using a JASCO-V 670 spectrophotometer (Tokyo, Japan) equipped with an integrating sphere for broadband antireflection analysis in the UV-Vis-NIR spectrum (Tokyo, Japan). The angular dependence of the specular reflectance of all GaAs samples was measured as a function of wavelength (250–2000 nm) and various AOIs (5–60 degrees) using a JASCO ARN-475 (Tokyo, Japan) spectral measurement accessory for broadband and wide-angle antireflection analysis in the UV-Vis-NIR spectrum. A stabilized helium–neon laser (632.8 nm) with a frequency stability of ±5 MHz was used to measure the specular reflectance of all GaAs samples as a function of the incident angle (30–85 degrees) for polarization-independent antireflection analysis at a wavelength of 632.8 nm.

### 2.3. Optical Modeling and Simulation

We hypothesize the presence of a GRI coating layer between the air and the bare GaAs substrate. Subsequently, we employed optical software (WVASE 32, J.A. Woolam) to generate two types of GRI profiles (linear and exponential sine [26]) and to simulate the s- and p-polarized reflectance data for these GRI profiles at various angles of incidence for a wavelength of 632.8 nm. The same software was also employed to simulate s- and p-polarized reflectance data for the surface of black GaAs samples. This fitting and the associated simulation were conducted under the assumption that the black GaAs samples have a GRI profile consisting of three graded layers. Details on the fitting of the graded index layers for black GaAs samples can be found in Supporting Table S1 and the accompanying description.

## 3. Results

### 3.1. Fabrication of Black GaAs NTs

**Figure 1:**
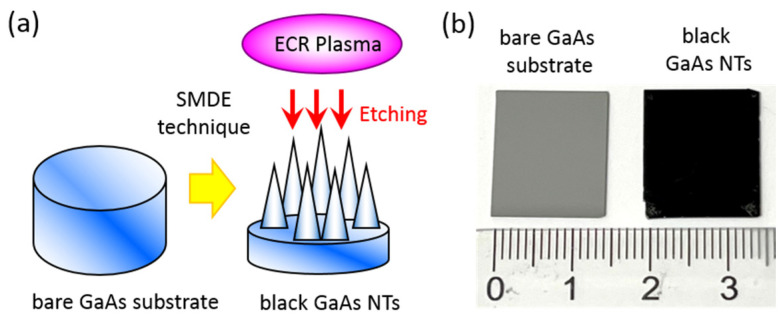
(**a**) A schematic illustration of the plasma process for fabricating black GaAs NTs on a bare GaAs substrate utilizing the self-masking dry etching method. (**b**) Photographic images depicting the bare GaAs substrate (**left**) and the GaAs substrate decorated with black GaAs NTs (**right**).

Figure 1a illustrates the one-step technique that was used to produce broadband, wide-angle, and polarization-independent antireflective nanostructures on bare GaAs surfaces through SMDE facilitated by high-density ECR plasma etching. This process involved creating specialized antireflective surface features to reduce reflection, thereby enhancing the optical absorption performance across a broad spectrum of wavelengths and angles of incidence. Figure 1b shows a comparison of photographs of a polished single-crystal GaAs substrate (left), which appears gray in color, and an etched GaAs substrate (right), which features antireflective nanostructures and appears to have a deep black color. This comparison highlights the significant antireflection properties of the latter surface.

**Figure 2:**
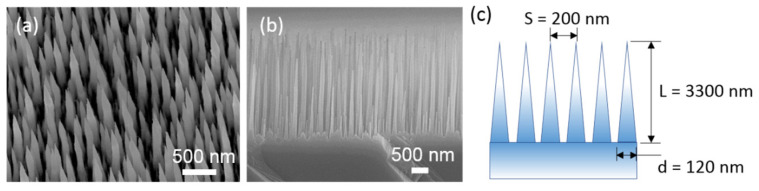
SEM images exhibiting a tilted top view (**a**) and a cross-sectional view (**b**) of black GaAs NTs having a length of 3300 nm. (**c**) Schematic diagram of the nanostructures and geometric parameters for black GaAs NTs. Here, L denotes the average length of the NTs, S represents the average spacing of the NTs, and d denotes the average diameter of the NTs.

Figure 2 shows the SEM images of surface morphology and geometric parameters of plasma-etched GaAs samples. The top view SEM image (Figure 2a) reveals that the GaAs nanostructured arrays are aperiodic in nature. Additionally, the cross-sectional SEM image (Figure 2b) shows that the aperiodic GaAs nanostructured arrays consist of numerous long and sharp needle tips (NTs). These GaAs NTs are geometrically characterized by an average diameter (d) of approximately 120 nm at the base, a distance between the NTs (spacing, S) of about 200 nm, and an average length (L) of about 3300 nm, as depicted in the schematic diagram in Figure 2c.

### 3.2. Broadband Antireflection Properties

**Figure 3:**
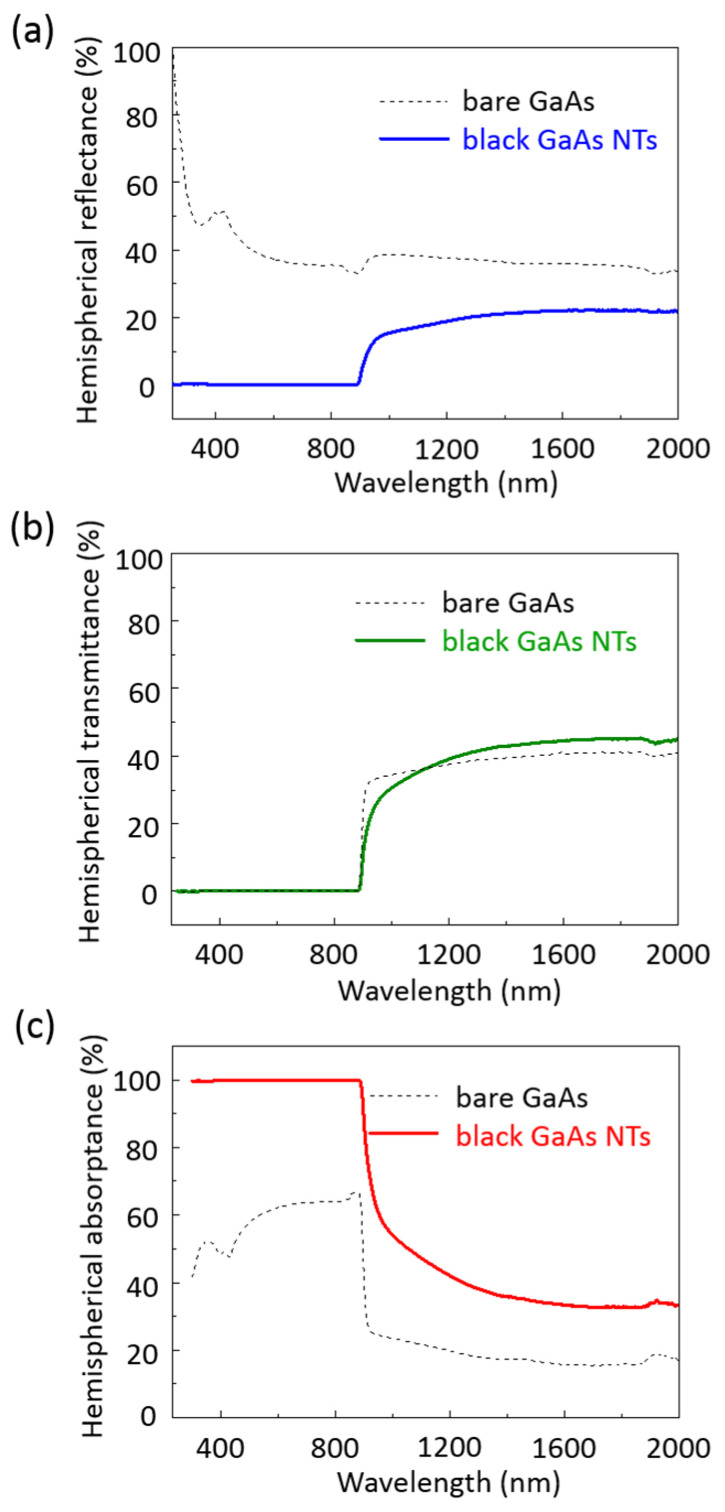
Broadband antireflection properties were observed on the surfaces of both bare GaAs and black GaAs NTs, spanning a wavelength range from 300 to 2400 nm. The hemispherical reflectance (**a**), hemispherical transmittance (**b**), and hemispherical absorbance (**c**) of bare GaAs (dashed line) and black GaAs NT (solid line) surfaces were examined.

We recorded broadband hemispherical reflection, hemispherical transmission, and hemispherical absorption spectra of black GaAs NT samples in the UV–visible–near-infrared region (300–2000 nm), along with bare GaAs substrates for reference (Figure 3). Figure 3a shows the hemispherical reflectance as a function of the wavelength for bare GaAs (black dashed line) and black GaAs NT (blue solid line) samples. The hemispherical reflectance of the untreated bare GaAs substrate reaches 39% in the visible–near-infrared region and exceeds 60% in the UV region. This high reflectivity is mainly due to the significant refractive index contrast between air and the GaAs dielectric medium (n = 3.8). Compared to the bare GaAs, the black GaAs NT samples show excellent antireflective capability over the full UV–visible–near-infrared region range (Figure 3a). The reflectivity of the black GaAs NT samples can be reduced significantly to between 0.3% and 0.1% in the UV–visible region, and approximately 22% in the NIR region (Figure 3a). Notably, our additional experimental results indicate that the length of the NT is a critical factor influencing its antireflective properties. As shown in Supporting Figure S1d, longer GaAs NTs exhibit superior antireflection performance compared to shorter GaAs NTs. Our experimental data clearly demonstrate that at a wavelength of 700 nm, increasing the nanotip length from 380 nm to 830 nm and further to 1100 nm results in a gradual decrease in specular reflection values, from 18% to 9% and finally to 5% (as shown in Supporting Figure S1e).

Figure 3b shows the hemispherical transmittance as a function of the wavelength for bare GaAs (black dashed line) and black GaAs NT (green solid line) samples. Sharp edges at 1.42 eV (λ = 873 nm) are seen in all GaAs samples, which can be ascribed to bandgap absorption. It is clearly observed that the transmittance of bare GaAs substrates increases from less than 1% at 1.42 eV (λ = 873 nm) to 42% at 0.62 eV (λ = 2000 nm) in the low-energy region. In the same region, the transmittance of the black GaAs NT samples is approximately 46%, which is slightly higher than that of the bare GaAs substrate.

Figure 3c shows the hemispherical absorptance as a function of the wavelength for bare GaAs (black dashed line) and black GaAs NT (red solid line) samples. First, the hemispherical absorbance (A) of all GaAs samples is calculated using the following formula:

A = 1 − R − T

where R represents the hemispherical reflectance and T represents the hemispherical transmittance. As shown in Figure 3c, the absorption spectrum of the bare GaAs substrate ranges from 240 nm to 873 nm, with absorption below 67% due to high reflectivity. In the wavelength region from 873 nm to 2000 nm, the absorption of the bare GaAs substrate is less than 30% due to both high reflectivity and transmittance. Black GaAs NT samples exhibit the highest light absorption in the region above the bandgap (250–873 nm), reaching up to 99%. Additionally, black GaAs NTs maintain a high absorbance of approximately 33% to 60% even when the incident energy is below the bandgap (873–2400 nm). Compared to the bare GaAs, the black GaAs NT samples exhibit excellent broadband antireflection properties across the entire UV/Vis/NIR region.

### 3.3. Broadband and Wide-Angle Antireflection Properties

**Figure 4:**
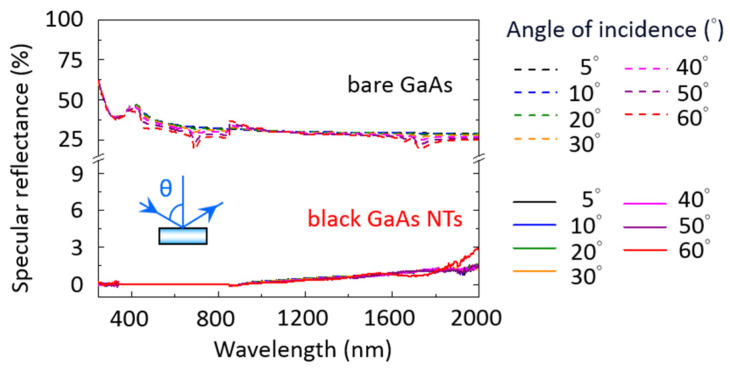
The angular-dependent specular reflectance as a function of wavelength (250 to 2000 nm) and various AOIs (5° to 60°) was measured for both bare GaAs (dashed line) and black GaAs NT (solid line) surfaces.

To achieve effective broadband and omnidirectional antireflection, the antireflective structure must exhibit low reflectance over a broad spectrum of AOIs. Accordingly, we analyzed the angular dependence of the specular reflectance of bare GaAs (dashed line) and black GaAs NT (solid line) samples across a wavelength range from 250 to 2000 nm and an AOI range from 5° to 60°. Figure 4 shows that the angular dependence of the specular reflectance of bare GaAs remains relatively constant across the range of AOIs from 5° to 60°, maintaining approximately 35–28% reflectivity within the visible–near-infrared wavelength range. The angular dependence of the specular reflectance of the black GaAs NT samples can be minimized to merely 0.3–0.1% in the UV–visible region. In the NIR region, it increases slightly to approximately 3% when the AOI is 60° (Figure 4). Compared to the bare GaAs sample, the black GaAs NT samples exhibit excellent angular-independent specular reflectance properties across the entire UV/Vis/NIR region and range of AOIs.

### 3.4. Optical Modeling for Gradient Refractive Index Coating

Light reflection occurs when light encounters the interface of two media with different refractive indices, changing its propagation route and returning to the original medium. It is well known that single-layer quarter-wavelength antireflective coatings can theoretically achieve zero reflection at a single wavelength. However, they cannot meet the requirements for broadband, omnidirectional, and polarization-insensitive antireflection. Another method of reducing reflectivity involves gradually lowering the refractive index of the film from that of the substrate to that of air, known as a GRI coating. Moreover, numerical calculations have demonstrated that single-layer GRI coatings with various refractive index profiles (e.g., linear [27], quintic [28], and exponential sine [29]) exhibit broadband, omnidirectional, and polarization-insensitive antireflective properties. GRI coatings can be single-layer, multi-layer, or surface-structured inhomogeneous films, characterized by a gradual change in the refractive index along the thickness gradient, making them ideal for reducing light reflectivity [23,30]. To gain a deeper understanding of the antireflective properties of black GaAs NTs, we studied the angle- and polarization-dependent reflectance analysis of GRI coatings and attempted to determine the actual GRI profile of black GaAs NT samples, a property that has not yet been reported.

**Figure 5:**
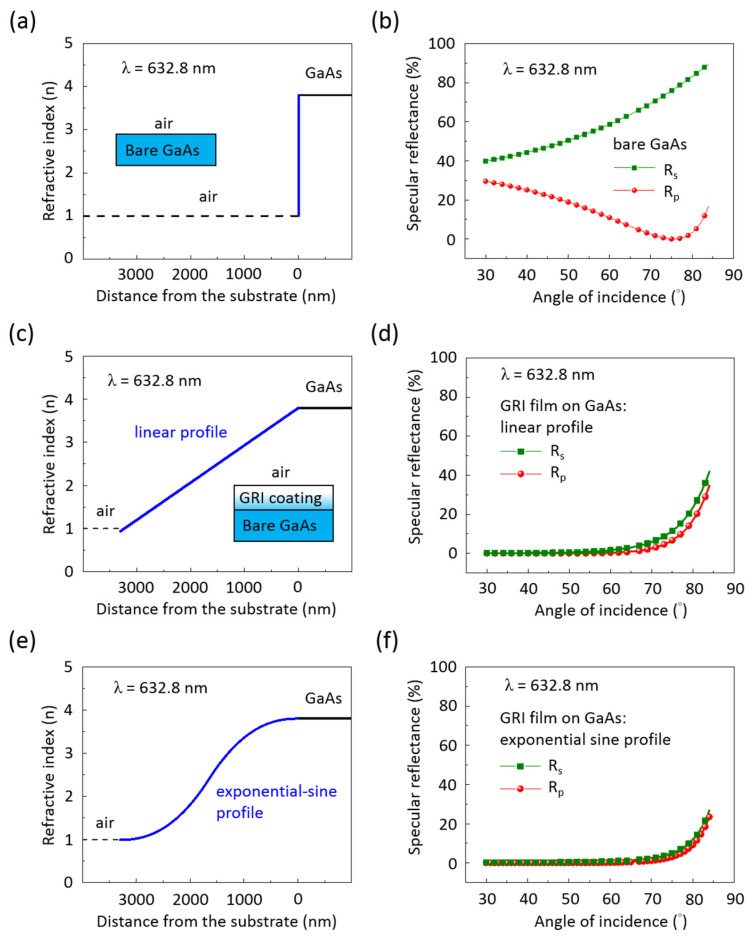
The establishment of a gradient refractive- index profile and subsequent simulation of angle-dependent reflectance data were conducted for bare GaAs with GRI coatings using various theoretical models: (**a**,**b**) only using the bare GaAs index profile, (**c**,**d**) using the linear index profile, and (**e**,**f**) using the exponential sine index profile. This process was carried out for s- and p-polarized light at a wavelength of 632.8 nm. S-polarized light has its electric field oriented perpendicular to the plane of incidence, whereas p-polarized light has its electric field oriented parallel to the plane of incidence.

We employed optical software WVASE 32 to create several numerical models for simulating the s- and p-polarized specular reflectance (Rs and Rp) across AOI values from 30° to 85° at a wavelength of 632.8 nm. These simulations were conducted for the air–GaAs interface using different GRI curve coatings, with the GRI coating thickness being 3300 nm, as shown in Figure 5. The numerical models of these GRI coatings encompass the refractive index profile of bare GaAs (Figure 5a,b), the linear profile (Figure 5c,d), and the exponential sine profile (Figure 5e,f). In Figure 5b, the Rs value for bare GaAs substrates increases as the AOI rises, while the Rp value initially decreases, reaching zero at the Brewster angle (~75°), and then increases with further increases in the AOI. When bare GaAs is coated with a GRI layer featuring a linear profile (Figure 5d), the Rs and Rp values show significant suppression compared to bare GaAs, maintaining low reflectance (<1%) with an AOI of up to approximately 60° for both s- and p-polarizations. In Figure 5f, both Rs and Rp values maintain low reflectivity even at an AOI of up to 70°, demonstrating improved reflectivity suppression performance when bare GaAs is coated with a GRI coating featuring an exponential sine profile.

### 3.5. Wide-Angle and Polarization-Independent Antireflection Properties

**Figure 6:**
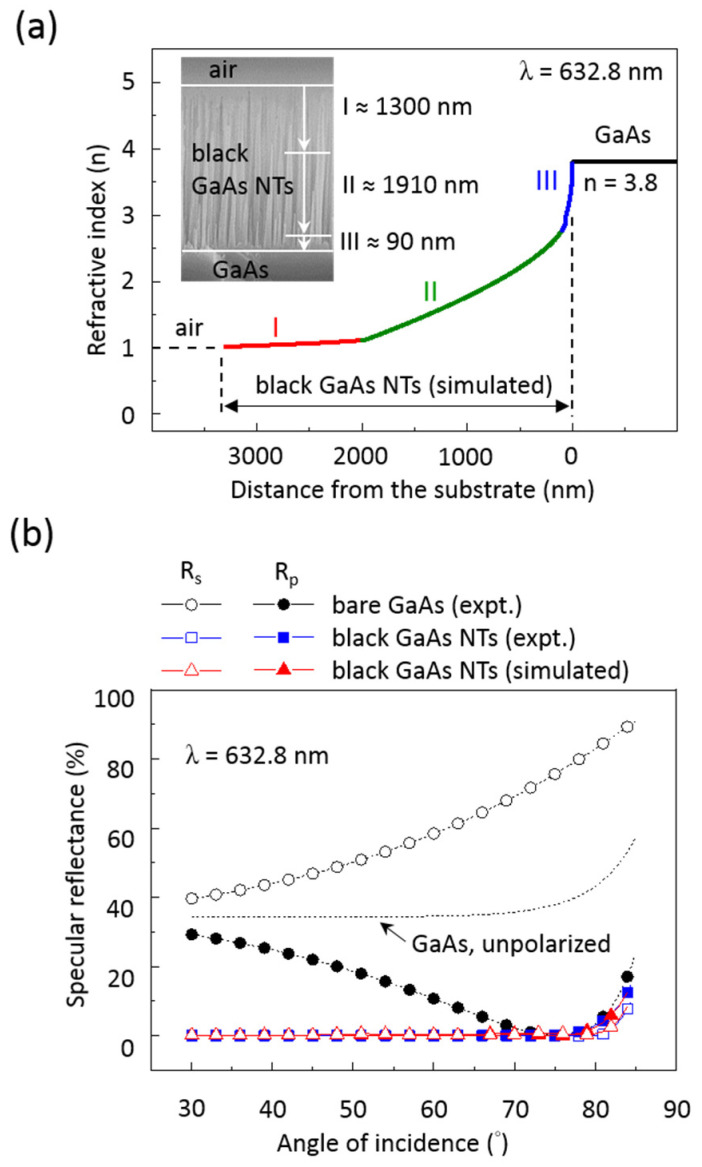
Simulation of the gradient refractive index profile of the black GaAs NT surface. (**a**) The refractive index profile of the black GaAs NT surface revealed three distinct regions: region I with a refractive index close to 1.0, region II exhibiting a graded refractive index, and region III with a refractive index near 3.8. The inset presents a cross-sectional SEM image of the black GaAs NTs, illustrating the three distinct regions. (**b**) The experimental and simulated specular reflectance of bare GaAs and black GaAs NT surfaces was evaluated by measuring their angle-dependent response using s- and p-polarized light at a wavelength of 632.8 nm. The unpolarized specular reflectance of bare GaAs surfaces has also been shown in the figure for reference.

To confirm the wide-angle and polarization-independent antireflection properties of black GaAs NTs, we analyzed and fitted the GRI profile, as shown in Figure 6. Using the same optical software, we simulated the optimal refractive index profile of the black GaAs NTs with a length of 3300 nm to fit the measured Rs and Rp data of the black GaAs NTs at a wavelength of 632.8 nm. The best-fit results indicate that black GaAs NTs are best modeled with a graded refractive index composed of three layers (region I, II, and III), rather than a single layer (Figure 6a). Region I is located near the top of the black GaAs NT and acts as the air–GaAs NT interface, extending approximately 1300 nm from the top. The GRI values for region I vary from n = 1.02 to 1.12. Region II spans 1900 nm from the bottom of region I, forming the bulk of the GaAs NT. The GRI values for region II range from n = 1.12 to 2.77. Region III is about 90 nm thick, positioned at the bottom of the GaAs NT, and serves as the GaAs NT-GaAs interface. The GRI values for region III are found to be from n = 2.77 to 3.8. The refractive index values for regions I and III are close to those of air (1.0) and GaAs (3.8 at 632.8 nm), respectively.

Figure 6b displays the measured Rs and Rp values (blue data points) as functions of the AOI for black GaAs NTs at a wavelength of 632 nm, accompanied by the simulated Rs and Rp values (red data points) across AOIs using GRI profiles from three distinct regions. Additionally, the measured angular reflectance data of bare GaAs (black data points) are also shown in Figure 6b. It is evident that the simulated Rs and Rp values derived from fitting the GRI curve for black GaAs NTs closely align with the experimentally measured Rs and Rp values for black GaAs NTs (Figure 6b). Additionally, both the experimental and simulated Rs and Rp data of black GaAs NTs exhibit apparent insensitivity to the AOI, contrasting with bare GaAs. This result demonstrates that black GaAs NTs with a GRI profile exhibit excellent wide-angle and polarization-independent antireflection properties. Therefore, black GaAs NTs can efficiently suppress Rs and Rp values (<1%) until the AOI exceeds 80°, at which point the angular reflectivity begins to increase.

## 4. Discussion

According to a numerical investigation based on Dobrowolski’s theory of optimal antireflection coatings, the modified quintic and exponential sine refractive index profiles from air to a substrate can exhibit superior wide-angle antireflective properties (up to approximately 89 degrees) compared to the original quintic and exponential sine refractive index profiles [26]. The primary distinction between the original quintic and exponential sine refractive index profiles is that the modified quintic and exponential sine profiles require a region with a low refractive index value, nearly equivalent to that of air. Furthermore, the thickness of this region with a low refractive index must constitute more than half, or even up to two-thirds, of the total GRI layer [26]. From this perspective, we can explain why black GaAs NTs with three layers of the GRI exhibit superior wide-angle and polarization-independent antireflective properties (AOI up to ~80°, as shown in Figure 6b) compared to a single layer with an exponential sine profile (AOI up to ~70°, as shown in Figure 5e,f). A reasonable explanation is that region I of the GRI profile for black GaAs NTs has a very low refractive index (n = 1.01 to 1.12), nearly equivalent to that of air, which is the lowest reported value to date, and constitutes approximately 40% of the total GRI thickness.

Notably, our previous studies [31], along with prevailing research on other materials [32,33,34,35] such as silicon nanotips, TiO_2_ and SiO_2_ nanorod layers, porous anodic alumina layers, grass-like alumina films, and porous amorphous germanium films, have shown that examining actual GRI profiles of antireflective structures is beneficial for understanding wide-angle and polarization-independent antireflective performance. To the best of our knowledge, the exploration of GRI profiles to verify antireflection performance has not been previously investigated in the context of black GaAs [6,7,8,9,10,11,12,13,14,15,16]. Our findings present the first demonstration of actual GRI profiles for black GaAs antireflective structures.

While longer GaAs antireflective structures achieve superior antireflective performance, especially polarization-independent properties, increasing the thickness of these nanostructures adversely affects electrical conduction from the standpoint of optoelectronic devices. Therefore, balancing the optical and electrical properties of GaAs antireflective structures is a significant scientific issue worth exploring when integrating them into optoelectronic devices in the future.

## 5. Conclusions

In conclusion, we have demonstrated the fabrication of broadband, omnidirectional, and polarization-independent antireflective black GaAs NTs on a bare GaAs substrate using the self-mask plasma etching process. The advantage of black GaAs NTs arises from their GRI profile, facilitating an order of magnitude reduction in hemispheric reflectance across a broad wavelength range, while concurrently enhancing hemispheric absorptance. Additionally, black GaAs NTs exhibited low specular reflectance over a wide range of AOIs for s- and p-polarized light. The notable antireflective properties exhibited across a broad spectrum of wavelengths and a wide angular range indicate that black GaAs NTs offer promising potential for enhancing the performance of diverse optoelectronic devices.

## Data Availability

Data are contained within the article and Supplementary Materials.

## Supplementary Materials

The following supporting information can be downloaded at: https://www.mdpi.com/article/10.3390/nano14131154/s1, Figure S1. Anti-reflection properties as a function of various lengths of GaAs NTs. SEM images of GaAs NTs with different lengths: (a) GaAs NTs with a length of 380 nm; (b) GaAs NTs with a length of 830 nm; (c) GaAs NTs with a length of 1100 nm. (d) Specular reflectance of bare GaAs and GaAs NTs with different lengths. (e) Specular reflection values at a wavelength of 700 nm for bare GaAs and GaAs NTs with different lengths. Table S1. Three graded refractive index layers of the black GaAs NT. Reference [36] are cited in the supplementary materials.
