# Supplementary material for: Plasma-Etched Black GaAs Nanoarrays with Gradient Refractive Index Profile for Broadband, Omnidirectional, and Polarization-Independent Antireflection
Source: Nanomaterials (Basel). 2024 Jul 6;14(13):1154. doi: 10.3390/nano14131154 (PMC11243535; doi:10.3390/nano14131154)
Supplement: Supplementary file 1 [file nanomaterials-14-01154-s001.zip › nanomaterials-3067762-supplementary.pdf]

Supplementary Information

Plasma-etched black GaAs nanoarrays with gradient refractive index profile for broadband, omnidirectional, and polarization-independent antireflection

Yi-Fan Huang[1*], Yi-Jun Jen[2], Varad A. Modak[3,4,5], Li-Chyong Chen[6,7,8], and Kuei-Hsien Chen[3,8]

[1]Department of Mechanical Engineering, National Chin-Yi University of Technology, Taichung 411030, Taiwan

[2]Department of Electro-Optical Engineering, National Taipei University of Technology, Taipei 106, Taiwan

[3]Institute of Atomic and Molecular Sciences, Academia Sinica, Taipei, 10617, Taiwan

[4]International Graduate Program of Molecular Science and Technology, National Taiwan University (NTU-MST), Taipei 10617, Taiwan

[5]Molecular Science and Technology Program, Taiwan International Graduate Program (TIGP), Academia Sinica, Taipei 11529, Taiwan

[6]Department of Physics, National Taiwan University, Taipei 10617, Taiwan

[7]Center of Atomic Initiative for New Materials, National Taiwan University, Taipei 10617, Taiwan

[8]Center for Condensed Matter Sciences, National Taiwan University, Taipei 10617, Taiwan

*Correspondence: yifanhuang@ncut.edu.tw

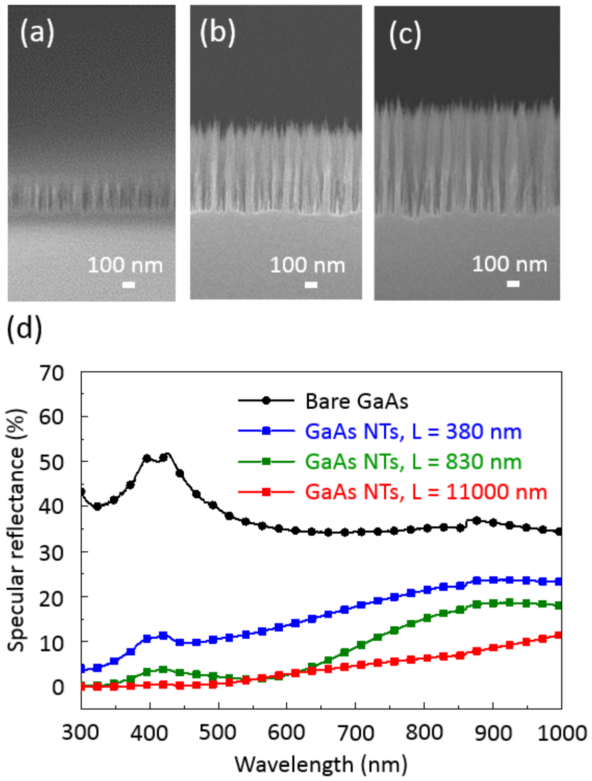

(e)

Sample	R% at a wavelength of 700 nm
Bare GaAs	35%
GaAsNTs with L= 380 nm	18%
GaAsNTs with L= 830 nm	9%
GaAsNTs with L= 1100 nm	5%

Supporting Figure S1. Anti-reflection properties as a function of various lengths of GaAs NTs. SEM images of GaAs NTs with different lengths: (a) GaAs NTs with a length of 380 nm; (b) GaAs NTs with a length of 830 nm; (c) GaAs NTs with a length of 1100 nm. (d) Specular reflectance of bare GaAs and GaAs NTs with different lengths. (e) Specular reflection values at a wavelength of 700 nm for bare GaAs and GaAs NTs with different lengths.

Supporting Table S1. Three graded refractive index layers of the black GaAs NT.

Interface of GRI	Layer number of GRI	GRI values
air-GaAs NT	Region I = 1300 nm	n= 1.02 to 1.12
bulk of GaAs NT	Region II = 1900 nm	n= 1.12 to 2.77
GaAs NT-GaAs interface	Region III = 90 nm	n= 2.77 to 3.8

The refractive index is calculated at a wavelength of λ = 632.8 nm.
The thickness and the GRI values for each layer are fitted according to the measured Rs and Rp data of the black GaAs NT at a wavelength of 632.8 nm using the WVASE 32 software.

The graded refractive index of black GaAs NTs was obtained by fitting the Rs and Rp data measured at a wavelength of 632.8 nm using WVASE 32 software. We found that using a single graded layer to fit the Rs and Rp data was unsuitable, regardless of the polynomial refractive index profile. Since black GaAs NTs are non-periodic nanostructures with varying nanotip lengths, there are different interfaces between the air and the GaAs NTs, including the air-GaAs NT interface, the bulk of the GaAs NT interface, and the GaAs NT-GaAs interface. Therefore, we constructed the graded layers of black GaAs NTs based on the dimensions observed from cross-sectional SEM images and the effective medium approximation concept [36], a method also reported in the literature for similar applications [32].
After fitting tests, we found that a graded refractive index profile with three layers fit the measured reflectance data better than profiles with one or two layers. While dividing black GaAs NTs into more than three layers can achieve similar fitting effects, our approach was to divide the entire thickness into three graded layers and then further subdivide each layer into finer layers, setting each 1 nm as a layer, with automatic fitting and solutions provided by the software (Fig. 6a). This method is relatively simple and effective. It is also important to note that these profiles were not derived from any polynomial equations (not a quintic curve) but through numerous divisions using the effective medium approximation (Fig. 6a).
